# PGNneo: A Proteogenomics-Based Neoantigen Prediction Pipeline in Noncoding Regions

**DOI:** 10.3390/cells12050782

**Published:** 2023-03-01

**Authors:** Xiaoxiu Tan, Linfeng Xu, Xingxing Jian, Jian Ouyang, Bo Hu, Xinrong Yang, Tao Wang, Lu Xie

**Affiliations:** 1 Department of Bioinformatics and Biostatistics, School of Life Sciences and Biotechnology, Shanghai Jiao Tong University, Shanghai 200240, China; 2 Shanghai-MOST Key Laboratory of Health and Disease Genomics & Institute of Genome and Bioinformatics, Shanghai Institute for Biomedical and Pharmaceutical Technologies, Shanghai 200237, China; 3 Liver Cancer Institute, Fudan University, Shanghai 200032, China

**Keywords:** neoantigen, noncoding regions, proteogenomics, prediction pipeline, tumor immunotherapy

## Abstract

The development of a neoantigen-based personalized vaccine has promise in the hunt for cancer immunotherapy. The challenge in neoantigen vaccine design is the need to rapidly and accurately identify, in patients, those neoantigens with vaccine potential. Evidence shows that neoantigens can be derived from noncoding sequences, but there are few specific tools for identifying neoantigens in noncoding regions. In this work, we describe a proteogenomics-based pipeline, namely PGNneo, for use in discovering neoantigens derived from the noncoding region of the human genome with reliability. In PGNneo, four modules are included: (1) noncoding somatic variant calling and HLA typing; (2) peptide extraction and customized database construction; (3) variant peptide identification; (4) neoantigen prediction and selection. We have demonstrated the effectiveness of PGNneo and applied and validated our methodology in two real-world hepatocellular carcinoma (HCC) cohorts. TP53, WWP1, ATM, KMT2C, and NFE2L2, which are frequently mutating genes associated with HCC, were identified in two cohorts and corresponded to 107 neoantigens from non-coding regions. In addition, we applied PGNneo to a colorectal cancer (CRC) cohort, demonstrating that the tool can be extended and verified in other tumor types. In summary, PGNneo can specifically detect neoantigens generated by noncoding regions in tumors, providing additional immune targets for cancer types with a low tumor mutational burden (TMB) in coding regions. PGNneo, together with our previous tool, can identify coding and noncoding region-derived neoantigens and, thus, will contribute to a complete understanding of the tumor immune target landscape. PGNneo source code and documentation are available at Github. To facilitate the installation and use of PGNneo, we provide a Docker container and a GUI.

## 1. Introduction

Neoantigens are considered to be promising therapeutic targets owing to their tumor specificity, and to their neither being affected by pre-existing immune tolerance nor generating autoimmunity [1]. Thus, neoantigens can be used as potential targets for therapeutic vaccines. A neoantigen vaccine is designed to trigger de novo T cell response and broaden the endogenous repertoire of tumor-specific T cells [2]. A phase-I trial of a neoantigen-based peptide vaccine showed that four patients with stage III melanoma induced CD4+ T cell and CD8+ T cell responses after vaccination and remained disease-free for a median follow-up of 25 months after vaccination. This demonstrates the potent tumor-specific immunogenicity and antitumor activity of neoantigen vaccines [3]. However, one major challenge in neoantigen vaccine design is the rapid and accurate identification of neoantigens that can induce T cell responses in patients. 

The advent of next-generation sequencing has provided opportunities to efficiently identify tumor-specific antigens in individual patients, leading to the exploration of clinical target therapies. In fact, several pipelines have been developed to predict neoantigens, such as pVACtools [4], NeoPredPipe [5], Neopepsee [6], etc. Although the development of these tools has opened the way for identifying potentially immunogenic neoantigens [7], limitations to these tools exist. First, most traditional prediction pipelines were developed based on genomic and transcriptomic data. Many false-positive neoantigens inevitably occur, due to the large number of mutations in individual patients and the limited performance of MHC ligand binding prediction [8]. In addition, studies have shown that the mRNA measurements of many genes correlate poorly with protein expression [9,10]. With advances in mass spectrometry (MS)-based proteomics, the combination of proteomics and genomics, i.e., proteogenomics, has been a major force in driving personalized neoantigen vaccine identification [11,12,13]. It allows the presence verification of those peptides that are most likely to generate an immune response based on neoantigen prediction pipelines; thus, such peptides may be moved into subsequent functional selection processes. Proteogenomics has greatly reduced the number of false positives for predicted neoantigens and has eased the burden of experimental validation. Our group previously developed proteogenomics neoantigen prediction pipelines, ProGeo-neo [14] and ProGeo-neo2.0 [15], and WEN B et al. developed NeoFlow [16]. 

Another limitation of the currently existing neoantigen prediction pipelines is that they almost all focus on genomic coding regions. While variants in protein-coding regions have received the most attention, numerous studies have noted the importance of noncoding variants in cancers [17]. Exomes only account for 2% of the human genome, whereas up to 75% of the genome has been shown to be transcribed and potentially translated [18]. Therefore, many allegedly noncoding regions are actually protein-coding. For example, long noncoding RNAs (LncRNA) are a type of noncoding RNA with a length of more than 200 nt, lacking a protein-coding function due to the lack of a complete open read frame (ORF) [19]. Intriguingly, several recent studies have noted LncRNAs as a source of new peptides [20,21]. Of particular relevance to tumor neoantigen discovery, 99% of cancer mutations are located in noncoding regions [17]. Therefore, focusing on the exome as the only source of tumor neoantigens is very restrictive. Notably, peptides derived from the noncoding regions have been shown to bind to MHC molecules, some of which were identified as the targets of T cells [22,23,24]. Subsequently, landmark studies demonstrated that the noncoding regions are the main source of targetable tumor-specific antigens [25,26]. However, an efficient and easy-to-use tool to predict and investigate the personalized neoantigens from noncoding regions is still lacking.

Herein, we present PGNneo, an integrated computational pipeline to predict noncoding neoantigens from RNA-seq and MS data. We demonstrated the effectiveness of PGNneo and validated our methodology in two real-world hepatocellular carcinoma (HCC) cohorts. In addition, we applied PGNneo to a colorectal cancer (CRC) cohort, demonstrating that the tool can be extended and verified in other tumor types. PGNneo is an efficient tool to identify noncoding neoantigens and can be easily installed and deployed at https://github.com/tanxiaoxiu/PGNneo. To be more user-friendly, we also provide a Docker version at (https://hub.docker.com/r/xiaoxiutan/pgnneo) and a GUI.

## 2. Methods

### 2.1. Data Collection

The paired-end sequencing data of lncRNA from 5 HCC patient-derived xenograft (PDX) samples, including tumor and normal tissues, were obtained from Hu et al. [27]. The proteomics datasets of the HCC cell line were downloaded from the ProteomeXchange Consortium (http://proteomecentral.proteomexchange.org) (accessed on 21 September 2020) with the identifier, PXD000529. This cohort is hereinafter referred to as HCC_HF. Another HCC cohort (hereinafter referred to as HCC_HT) from a previous collaboration with Jiang et al. [28] included RNA-seq files and MS raw data from 10 patients (early stage of HCC, subtype 3) were downloaded from the Gene Expression Omnibus (GEO) (accession number GSE124535) (accessed on 15 November 2021) and iProX database (http://www.iprox.org, accession number IPX0000937000) (accessed on 15 November 2021), respectively. Detailed sample information is provided in Table S1 in the Supplementary Materials. In addition, we collected a CRC cohort [29], including RNA-seq data and MS raw data from three CRC cell lines and RNA-seq data from one normal fetal small intestine cell line. This can be downloaded from the GEO (accession number GSE195985) (accessed on 2 October 2022) and the ProteomeXchange Consortium (Identifier PXD028309) (accessed on 2 October 2022), respectively. Detailed sample information of this cohort is presented in Table S12 in the Supplementary Materials. 

The human reference genome (hg38) and Proteome (version 101) were downloaded from UCSC (http://hgdownload.cse.ucsc.edu/goldenPath/hg38/bigZips/hg38.fa.gz) (accessed on 1 September 2020) and the Ensembl database (http://www.ensembl.org/) (accessed on 1 September 2020), respectively. Contaminated protein sequences were available in a FASTA format from the common repository of adventitious proteins (cRAP) (http://www.thegpm.org/crap/) (accessed on 1 September 2020).

### 2.2. RNA-Seq Data Processing

RNA-seq raw data were cleaned by Trimmomatic (v0.39) (Max Planck Institute of Molecular Plant Physiology, Golm, Germany) [30], with the standard adapters trimmed and low-quality reads filtered. All clean reads were aligned to the human reference genome using the Burrows–Wheeler alignment tool (BWA, v0.7.17) (Wellcome Trust Sanger Institute, Cambridge, UK) [31] with the default parameters. The resulting .sam file was converted to .bam, sorted, and indexed using samtools [32]. The Picard [33] tool, MarkDuplicates (Broad Institute, Cambridge, MA, USA), was used to identify duplicates. To correct as many systematic errors in the sequencing process as possible, we performed base quality score recalibration. The Picard AddorReplacereAdgroups tool (Broad Institute, Cambridge, MA, USA) was used to modify the headers of BAM files for subsequent processing. Somatic single nucleotide variants (SNVs), and insertions and deletions (Indels), were detected by GATK Mutect2 (v4.1.9) (The Broad Institute of Harvard and MIT, Cambridge, MA, USA) [34] from the BAM files of paired tumor and normal samples. The GATK FilterMutectCalls (The Broad Institute of Harvard and MIT, Cambridge, MA, USA) tool was used to filter somatic mutations using default parameters, with true positive mutations marked with “PASS” and we selected “PASS” mutations. 

Since affinity predictions for the peptide-MHC interface are MHC-specific, it is critical to know the patient HLA types. HLA alleles in each sample were inferred from trimmed RNA-seq data using OptiType (University of Tübingen, Tübingen, Germany) with the default settings, which has been demonstrated to achieve HLA typing with ~97% accuracy [35,36].

### 2.3. Mutation Annotation and Peptide Extraction

**Figure 1:**
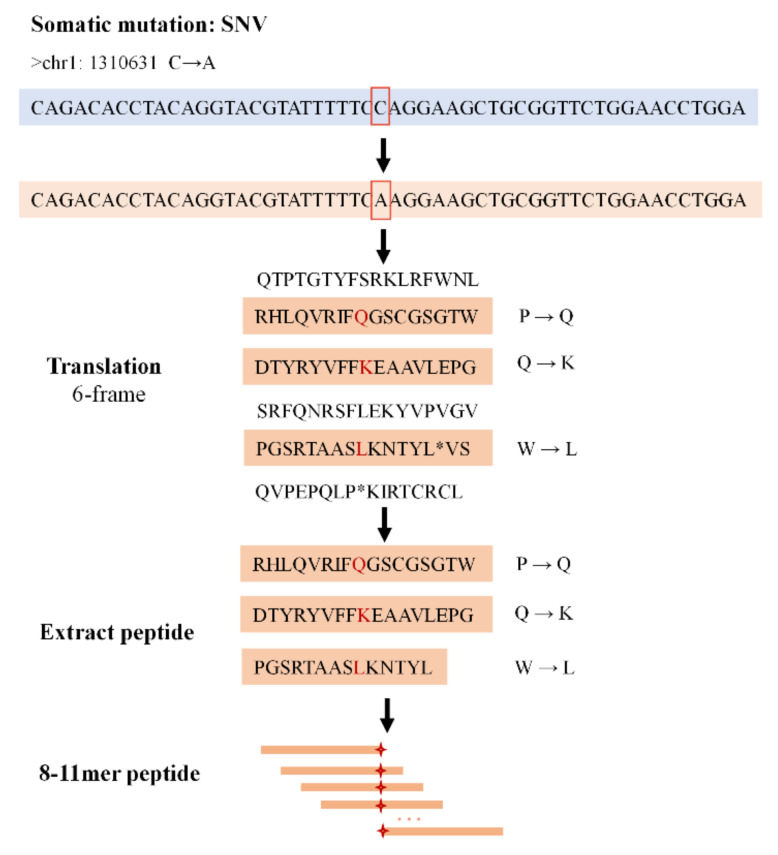
Generation of the variant peptides.

The annotation of mutations and the extraction of the peptide are shown in Figure 1. Somatic mutation data that were obtained based on RNA-seq data were annotated using Annovar [37] to identify noncoding-region somatic mutations, including SNVs and indels. The noncoding regions that we used specifically included: “downstream”, “intergenic”, “intronic”, “ncRNA_exonic”, “ncRNA_intronic”, “ncRNA_splicing”, “splicing”, “upstream”, “UTR3”, and “UTR5”. After mutation filtering, nucleotide sequences with a set interval length were obtained according to the mutation sites and reference genome using Bedtools (University of Virginia School of Medicine, Charlottesville, VA, USA) [38]. Specifically, 100 nucleotide sequences were taken from along each side of the mutation site. To generate the nucleotide sequences containing the mutation, we replaced the reference bases with mutant bases, i.e., we replaced the “REF” column with the “ALT” column in the mutation table.

For the translation of nucleotide sequences in the genome, six-frame translation is the classical method. Six-frame translation has the advantage of being independent of any a priori annotation of the nucleotide sequence [39,40]. Thus, according to the 64 codons, the mutant nucleotide sequences were translated into novel proteins via six-frame translation. The termination codons were replaced with “*” and the protein sequences were cleaved into short peptides according to “*”. The short peptides that did not contain the mutations were then filtered out. Finally, we obtained tumor protein sequences containing mutations from noncoding regions.

### 2.4. Database Construction and Peptide Identification

Identifying a mutant peptide expressed at the protein level is a crucial step. In this study, MaxQuant (Max-Planck Institute for Biochemistry, Martinsried, Germany) [41], a proteomics identification quantitative tool, was used to identify the peptides. To search the proteomics data, we first constructed a customized database for each individual tumor sample, including human reference proteins, common contaminant protein sequences in the laboratory (cRAP), and cancer-specific proteomes.

Then, to filter for true peptides, all MS/MS spectra were searched using MaxQuant in the customized peptide database. A separate target-decoy search strategy was used. Decoy peptides were generated from the peptides of corresponding target databases using a reversed tryptic approach. The parameters of MaxQuant were set as follows: (1) the variable modifications included protein N-terminal acetylation with methionine oxidation; (2) strict trypsin specificity was required, allowing up to two missed cleavages; (3) the carbamidomethylation of cysteine was set as a fixed modification. In addition, false discovery rate (FDR) thresholds for protein peptides were specified at 1%. The minimum required peptide length was set to 7 amino acids. Finally, we extracted the cancer-specific mutant peptides identified by MS data and provided evidence in terms of protein expression level. 

### 2.5. Neoantigen Prediction and Selection

PGNneo uses NetMHCpan 4.1 (Department of Bio and Health Informatics, Technical University of Denmark, Kgs. Lyngby, Denmark) [42] to calculate the binding affinity of peptides to patient-specific HLA alleles. To match the length of the peptides bound by MHC-I molecules, the peptides that were filtered by mass spectrometry were cleaved into short peptides containing mutated 8–11 mers. The percentile rank score was proven to exhibit higher sensitivity and less bias in HLA-peptide identification than the half-maximum inhibitory concentration (IC50) [43]. Therefore, the percentile rank (%rank) value was used as the metric of HLA-peptide binding prediction, and peptides with a %rank < 2 were considered to be candidate neoantigens. Similar sequences often originate from a common ancestral sequence and they are likely to have similar spatial structure and biological function; in fact, tumor-infiltrating T cells were found to exhibit a cross-reactivity that recognizes both tumor neoantigens and homologous non-tumor microbial antigens [44]. Therefore, to filter candidate neoantigens, sequence similarity analysis was performed using the basic local alignment search tool (BLAST) (National Center for Biotechnology Information, Bethesda, MD, USA) [45]. In total, 746 experimentally immunogenic neoantigens, collected from an in-house database, dbPepNeo2.0 [46], were used to build the target sequence database, while candidate neoantigens were treated as retrieval sequences. Then, BLASTp was used to identify homologous sequences so that the degree of homology between candidate neoantigens and immunogenic neoantigens could be established. We adjusted several default options to increase the sensitivity of BLAST searches that were performed with short input sequences. The peptides were reported to have sequence identity to immunogenic neoantigens if the percentage of identical matches was above 60%. 

### 2.6. PGNneo Pipeline Implementation

PGNneo is open-source software written in Python, shell, and R. The software is divided into four toolkits, based on four modules. The user needs to configure the path and parameters of the software before applying the toolkits. After completing the configuration, the pipeline can be run by executing the command line. A more detailed tutorial on the use of PGNneo is available in the User’s Manual. The PGNneo source code and documentation are available at https://github.com/tanxiaoxiu/PGNneo. To facilitate the installation and use of PGNneo, we provide a Docker container (Docker: https://hub.docker.com/r/xiaoxiutan/pgnneo) and a GUI.

## 3. Results

### 3.1. The Workflow of the PGNneo Pipeline

**Figure 2:**
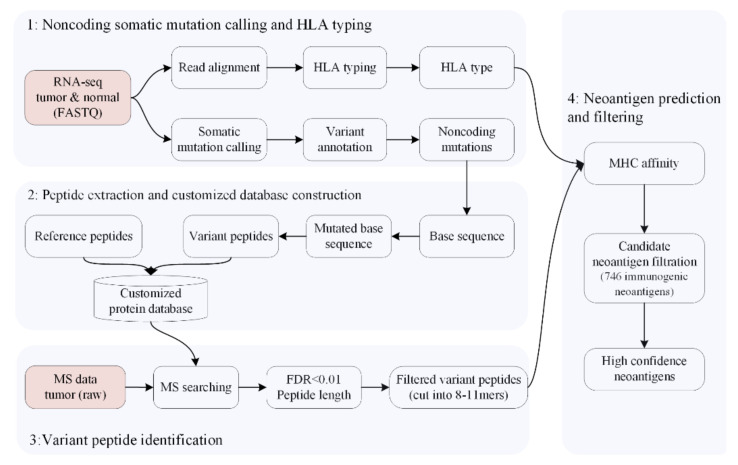
Overview of the PGNneo pipeline for proteogenomics-based noncoding neoantigen prediction.

Here, as shown in Figure 2, a versatile and comprehensive workflow, PGNneo, is presented to identify neoantigens in the noncoding region. In PGNneo, several input datasets are required, including RNA-seq profiles and MS datasets. First, the RNA-seq profiles from the paired tumor and normal samples are used to screen for somatic mutations in the noncoding regions, and the amino acid sequences containing mutant sites in the noncoding region can thus be identified. Then, those expressed sequences can be filtered using MS datasets. Eventually, the resulting peptides are used for neoantigen prediction and selection by using MHC affinity and the database dbPepNeo2.0 [46]. 

The general computational framework of PGNneo consists of the following modules. (1) Noncoding somatic variant calling and HLA typing. Identifying somatic mutations in tumor cells is a key step in the neoantigen presentation pathway. For this purpose, paired tumor and normal samples were used for somatic variant calling. Prior to annotation, we removed any low-quality somatic mutations. Eventually, the noncoding mutations were extracted. The prediction of HLA typing was performed, based on the RNA-seq data from tumor samples. (2) Peptide extraction and customized database construction. Working according to the mutation information, the nucleotide sequences were obtained and were then translated into proteins by six-frame translation. The process of extraction of the peptide is shown in Figure 1. Eventually, tumor mutant peptides were obtained. Subsequently, these mutant protein sequences and reference proteins were combined to construct a customized protein database. (3) Variant peptide identification. The resulting peptides were filtered using MS datasets. The proteomic data provided evidence not only for the presence of peptides at protein levels but also for the binding of peptides to MHC molecules. (4) Neoantigen prediction and selection. Candidate neoantigens were predicted, according to peptides and HLA types, using NetMHCpan 4.1. The candidate neoantigens were filtered using the database dbPepNeo 2.0, which is an in-house dataset using 746 experimental immunogenic peptides as a reference. The resulting datasets at different filtering stages could then be obtained and downloaded according to the user’s preference. Table 1 summarizes the software used in PGNneo.

### 3.2. Evaluation of PGNneo Pipeline Results

**Figure 3:**
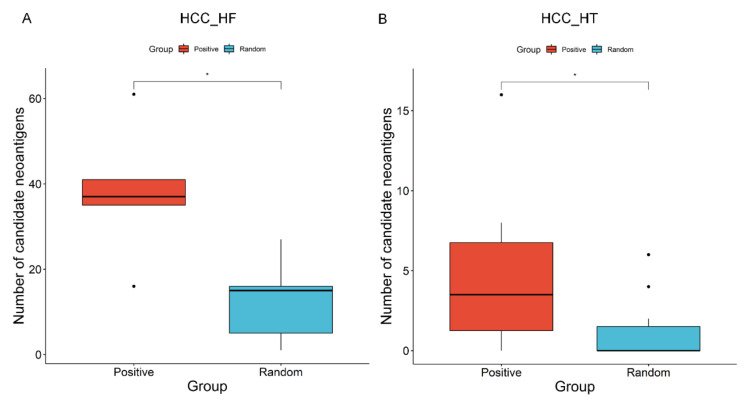
Evaluation of candidate neoantigens predicted by PGNneo, based on independent datasets: boxplot of the positive and random peptides in the HCC_HF cohort (**A**) and the HCC_HT cohort (**B**), with a * *p*-value < 0.05, ascertained by a Wilcoxon test.

To evaluate the performance of PGNneo, we performed sequence similarity analysis using two independent datasets. The positive dataset comprises 746 experimentally validated immunogenic neoantigens (Positive) collected from the dbPepNeo2.0 dataset, and the background control dataset contains 6400 mutant peptides (Random) of length 8–11 residue [48]. We performed sequence similarity analysis on the candidate neoantigens (unfiltered) that were obtained from the two cohorts of HCC_HF and HCC_HT with the positive dataset and random dataset, respectively. Two sequences exhibiting more than 60% of identical matches and an E-value of less than 0.5 are considered to be similar. Figure 3 shows that the results obtained from the positive and random datasets were significantly different in both the HCC_HF and HCC_HT cohorts, with *p*-values of 0.0278 (<0.05) and 0.01704 (<0.05), respectively (Wilcoxon test). The results indicate that the candidate neoantigens predicted by our method are more likely to have immunogenic potential. Therefore, filtering using the positive datasets was incorporated into the pipeline. This can be considered as an in silico verification step in the pipeline for the prediction of neoantigens.

### 3.3. Neoantigen Prediction, Selection, and Cross-Comparison from HCC Cohorts

**Figure 4:**
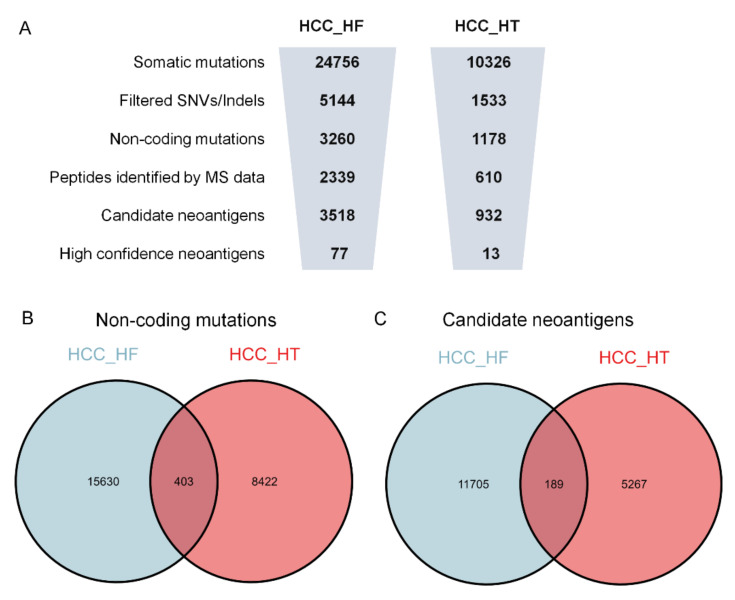
Results overview for the neoantigen discovery process. (**A**) The average number of key steps in the PGNneo on the HCC_HF cohort and HCC_HT cohort, respectively. (**B**) Overlapping non-coding mutations in the HCC_HF cohort and the HCC_HT cohort. (**C**) Overlapping candidate neoantigens in the HCC_HF cohort and the HCC_HT cohort.

PGNneo was applied to two independent HCC cohorts. We statistically analyzed the number of key steps in the pipeline for each sample **(**Figure S1 in the Supplementary Materials). In the HCC_HT cohort, one sample deviated significantly from other samples, possibly due to data quality problems, so this sample was deleted; finally, for this cohort, 9 samples were retained. The average number of key steps in the pipeline on the HCC_HF and HCC_HT cohorts are shown in Figure 4A. After filtering and annotation, an average of 3260 and 1178 noncoding mutations were obtained (Tables S2 and S3 in the Supplementary Materials). At the protein level, an average of 2339 and 610 peptides were identified by MS data analysis. HLA genotypes were predicted from the RNA-seq fastq file using the Optitype, and MHC-I binding predictions for the filtered peptides were predicted with netMHCpan4.1. As a result, an average of 3518 and 932 candidate neoantigens were obtained in the two cohorts, respectively (Tables S4 and S5 in the Supplementary Materials). After screening for HCC_HF, an average of 77 noncoding high-confidence neoantigens were eventually identified in each sample, ranging from 37 to 147 (Table S6 in the Supplementary Materials). However, in the HCC_HT cohort, an average of 13 noncoding high-confidence neoantigens were identified per sample, ranging from 4 to 23 (Table S7 in the Supplementary Materials). Upon comparing the results of the two cohorts, we found 403 overlapping non-coding mutations and 189 overlapping candidate neoantigens in the two cohorts (Figure 4B,C); however, no overlap was found in the high-confidence neoantigens. The results show that neoantigens are unique and the number of neoantigens varies greatly between different datasets. 

**Figure 5:**
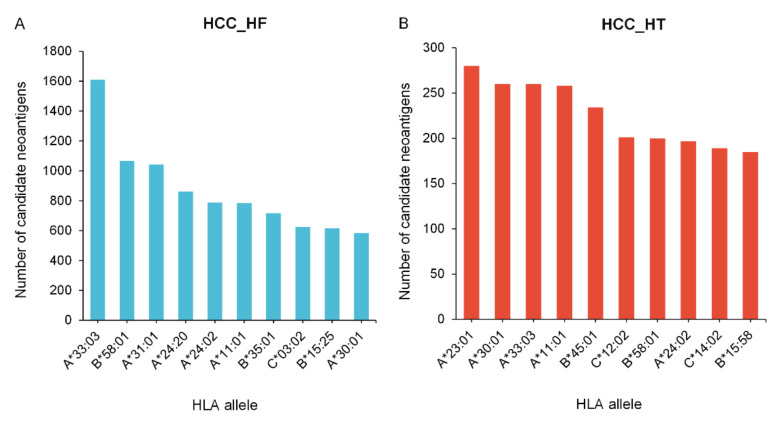
The number of predicted neoantigens binding with each HLA allele. (**A**) The top 10 HLA alleles matched with candidate neoantigens in the HCC_HF cohort. (**B**) The top 10 HLA alleles matched with candidate neoantigens in the HCC_HT cohort.

In addition, 26 and 28 unique HLA alleles were predicted for the two cohorts, respectively (Table S8 in the Supplementary Materials). We further calculated the frequency of HLA alleles in the sample population using the getHlaFrequencies function in the midasHLA package of R [49]. Then, the mean value of neoantigens bound by each HLA allele was calculated, based on the count of HLA alleles, thus normalizing the number of neoantigens. The frequency of HLA alleles and the number of corresponding neoantigens are given in Table S9 in the Supplementary Materials. Based on the ranking of the normalized number of neoantigens, the 10 most frequent binding HLA alleles that matched with candidate neoantigens in two cohorts are shown in Figure 5A and Figure 5B, respectively. The results showed that HLA alleles exhibited a preference for the binding of neoantigens, while HLA-A33:03 and HLA-A23:01 accounted for the largest binding proportion in the HCC_HF and HCC_HT cohorts. Moreover, the same candidate neoantigen can bind to different HLA alleles; this neoantigen is more likely to be present and may be applicable to a wider range of individuals.

### 3.4. The Sharing of Noncoding Neoantigens and Genes in Different Samples 

We further analyzed the overlapping neoantigens and their corresponding genes in the two cohorts. In the HCC_HF cohort (lncRNA-seq data from 5 HCC PDX samples and MS data from the HCC cell line), 10 neoantigens were found to be in common in at least 2 patients (Table S10 in the Supplementary Materials). These overlapping neoantigens are called “shared neoantigens” and have the potential to be designed as shared neoantigen vaccines. Conversely, neoantigens across the 5 patients were mapped to 118 unique genes, and 6 of these genes were observed in at least 2 patients (Table S10 in the Supplementary Materials). These overlapping genes are “hot-spot mutations” where the corresponding neoantigens may be in common among multiple patients. Unfortunately, no overlapping neoantigens or genes were found in the HCC_HT cohort (with paired RNA-seq data and MS data from 10 human HCC samples). This may be because the HCC_HF cohort dataset comprises unpaired lncRNA-seq data and MS data; the MS data is cell line data with lower heterogeneity, so that shared neoantigens can be found, while the HCC_HT cohort is of paired RNA-seq data and MS data from patients with a higher degree of individualization. To some extent, this explains the individualization of neoantigens in real patients. Therefore, this also reinforces the necessity for more research on hot-spot mutations for building up data resources for shared neoantigens.

### 3.5. Function Verification Analysis of Frequently Mutated Genes and Neoantigens in HCC

In order to correlate the predicted neoantigens with the clinical information garnered from patients, we explored the association between the predicted neoantigens and the pathogenesis of HCC. Rao et al. [50] summarized the frequently mutated genes and their functions that are associated with HCC. We compared candidate neoantigen-associated genes in the HCC_HF cohort and HCC_HT cohort with the frequently mutated genes associated with HCC. The TP53, WWP1, ATM, KMT2C, and NFE2L2 mutant genes associated with HCC were identified in two cohorts and corresponded to 98 neoantigens (Table S11 in the Supplementary Materials). Table 2 only shows information about the 26 candidate neoantigens that bind most strongly to HLA. Among them, TP53 is one of the most studied tumor suppressors, with multiple functions, and is associated with DNA damage checkpoints and repair defects. WWP1 is associated with the activation of oncogenic pathways in HCC; the overexpression of WWP1 promotes tumorigenesis in HCC patients and predicts poor prognosis. The loss of ATM reduces hepatocyte apoptosis and fibrosis, suggesting that the activation of ATM in response to oxidative stress plays a role in hepatic fibrosis development. KMT2C and KMT2D are functionally similar and may be involved in chromatin localization and genomic instability. NFE2L2 deficiency may render cells susceptible to oxidative stress-mediated DNA damage. Genes that are highly mutated in HCC may be attractive potential therapeutic targets.

### 3.6. Extended Application of PGNneo to Other Tumor Types

In addition, we applied PGNneo to another tumor type with moderate TMB, colorectal cancer (CRC) [29]. Firstly, 4206, 3664, and 5823 non-coding mutations were obtained on COLO205, SW620, and HCT116 cell line data, respectively, and further predictions yielded 217, 330, and 291 candidate neoantigens. In addition, to evaluate the potential immunogenicity of candidate neoantigens, we obtained high-confidence neoantigens based on the filtering of an experimentally validated immunogenic neoantigen database constructed by our group. Detailed results on sample information, noncoding region mutations, HLA typing, candidate neoantigens, and high-confidence neoantigens are provided in Table S12 in the Supplementary Materials. The results demonstrate that our pipeline can be applied to multiple tumor types. The biological mechanisms of noncoding neoantigens may be cross-verified as the application of PGNneo expands.

### 3.7. Comparing PGNneo with Other Tools

**Figure 6:**
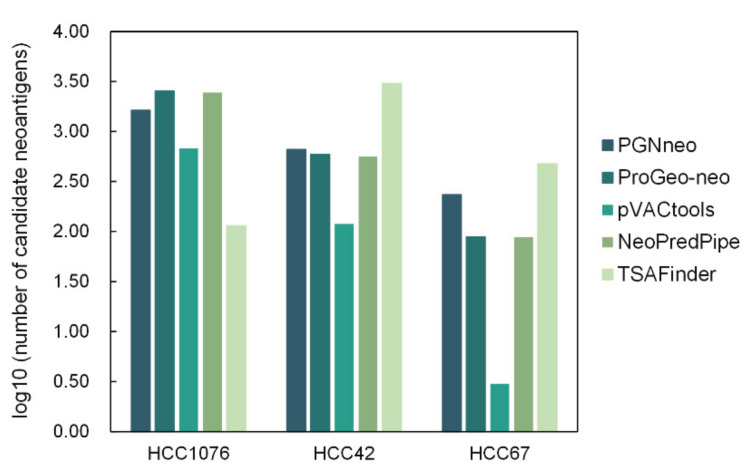
The number of candidate neoantigens predicted by PGNneo, ProGeo-neo, pVACtools, NeoPredPipe, and TSAFinder.

To complete the identification and comparison of neoantigens from both coding and noncoding regions, we applied four other common neoantigen prediction tools, including ProGeo-neo [14], pVACtools [4], Neopredpipe [5], and TSAFinder [51], to compare their performance with our own tool, PGNneo. pVACtools, Neopredpipe, and TSAFinder require fastq data for RNA-seq and/or VCF data for mutations as input, and ProGeo-neo requires the additional input of MS data. For two HCC cohorts, we randomly selected three patient samples, HCC42, HCC67, and HCC1076, for comparison across five neoantigen prediction tools. Since most neoantigens are composed of 9 amino acids, we only compared the prediction of 9-mer neoantigens [52]. The number of candidate neoantigens obtained by the five tools is shown in Figure 6. It is worth noting that PGNneo introduces MS data and shows candidate neoantigens after MS filtering so that the results of PGNneo are more stringent. Although ProGeo-neo also has a module for MS data filtering, unfortunately, no neoantigens were obtained after MS data identification. This is consistent with studies on neoantigens in the coding region of HCC, which suggested that the tumor mutational burden (TMB) of HCC is relatively low and neoantigens in coding regions are scarce [53]. In contrast, the other tools do not have an MS filtering step, and we only show their candidate neoantigens as predicted by peptide-MHC binding affinity (Table S13 in the Supplementary Materials). 

In addition, PGNneo sets up a module for secondary filtering by using 746 experimentally validated neoantigens, resulting in high-confidence neoantigens. Twenty, three, and one high-confidence 9-mer neoantigens were obtained in samples HCC1076, HCC42, and HCC67, respectively (Table S13 in the Supplementary Materials). Furthermore, we explored the association between these high-confidence neoantigens and the pathogenesis of HCC. The high-confidence neoantigen genes TNFSF14, GAD1, STARD1, and DHRS4-AS1 have been reported in the literature to be closely associated with HCC [54,55,56]. Specifically, TNFSF14 and GAD1 are highly expressed in HCC [54]; STARD1 promotes the progression of non-alcoholic steatohepatitis to HCC via bile acids [55]; DHRS4-AS1 ameliorates HCC by suppressing proliferation and promoting apoptosis via the miR-522-3p/SOCS5 axis [56].

Moreover, we analyzed the overlap of the candidate neoantigens predicted by different tools. For three samples, HCC1076, HCC42, and HCC67, the number of neoantigens identified by at least three tools was 411, 80, and 3, respectively (Table S14 in the Supplementary Materials). We recommend using multiple tools to predict neoantigens, which may yield more reliable results. Our investigation demonstrates that for cancer types with a low TMB, the source of neoantigens may be enriched when the noncoding region is taken into consideration. Therefore, PGNneo aims to expand the scope of neoantigen prediction and provide a richer neoantigen reference for some cancer types with a low TMB in the coding region.

## 4. Discussion

Although some algorithms and tools have been developed to tackle the problem of neoantigen prediction, most are based on coding regions. However, in the human genome, 98% of the sequence involves noncoding regions, and most DNA sequence variants occur in the noncoding regions [17]. The general properties of sequence variants are also applicable to noncoding variants, such as SNVs and Indels. What is more, noncoding variants can also generate neoantigens. However, there are few prediction tools that operate on noncoding regions; the majority of tools focus on exonic variant calling, which is based on genomic data rather than transcriptomic data. For this reason, we developed a proteogenomics-based pipeline, PGNneo, to identify those neoantigens derived from noncoding regions, based on transcriptomic data from the human genome. Furthermore, we successfully validated the effectiveness of PGNneo through its application in two HCC cohorts. A total of 386 and 113 high-confidence neoantigens were identified in the two HCC cohorts, respectively. In addition, we applied PGNneo to a CRC cohort, demonstrating that the tool can be extended to multiple tumor types.

Compared with the traditional neoantigen prediction pipeline, PGNneo has several advantages. First, it focuses on neoantigens in noncoding regions, which provides a new source of neoantigens for low-TMB tumor types. Studies have shown that for cancer types with a low TMB, such as liver cancer, the source of neoantigens should be extended to noncoding regions for better applicability to immunotherapy [53]. Second, it combines transcriptomics and proteomics data, furthering the proteogenomics neoantigen prediction pipelines for coding regions in our research group [14,15]. Most of the previously developed neoantigen prediction tools are based on genomic data only, and the predicted false-positive rate of neoantigens is relatively high. Combined with proteomic data, the accuracy of neoantigen prediction can be improved. Moreover, proteogenomics holds the promise of providing deeper mechanistic insights to enable the better matching of patients to targeted therapies than when analyzing each type of omics data separately. In addition, our pipeline uses RNA-seq data from paired tumor and normal tissues to call somatic mutations. Mutations in the RNA-seq data provide a better reference for a proteomics dataset than WES, mainly because of the ability of RNA-seq to identify novel somatic variants, while for oncogenes that are highly expressed in cancer, RNA-seq provides higher sequencing coverage than WES and, therefore, has higher statistical confidence in detecting variants [57]. Thus, in our pipeline, the customized searchable peptide database was derived from tumor RNA-seq data.

Compared to the coding-region-based proteogenomic prediction pipeline established by our group, in terms of data requirements, ProGeo-neo requires data at the genomic, transcriptomic, and proteomic levels of the patients, while PGNneo only requires transcriptomic and proteomic data from patients, and not WES/WGS data. This is because the neoantigen prediction step in ProGneo-neo is performed based on the mutations detected by WES/WGS, whereas in PGNneo, the step is based on RNA-seq data. Therefore, the application scenario of the PGNneo can be further expanded. 

There are still some limitations to our study, so we will expand on the following aspects. To further enrich the types of neoantigens, we may later update the tool to predict those neoantigens derived from gene fusion and RNA splicing, which will provide more potential neoantigen targets for developing therapeutic cancer vaccines. In addition, considering the complementary roles of coding-region-based and noncoding-region-based pipelines in identifying neoantigens, we will integrate neoantigen prediction tools such as ProGeo-neo that have already been developed within the group to further facilitate their use by researchers. Moreover, as noncoding neoantigens have been shown to have strong tumor specificity, more relevant studies have since emerged in the field. Recently, Cai et al. [58] developed IEAtlas, an atlas of HLA-presented immune epitopes derived from noncoding regions, which provides a valuable data resource for studying the immunogenicity of noncoding epitopes. Therefore, we will combine the data from IEAtlas to further improve the predictive power of PGNneo. Even though the subsequent experimental validation of candidate neoantigens is essential for real-world clinical application, our computational methods can narrow down the range of neoantigens to a certain extent and thereby pave the way to improved preclinical vaccine design. Therefore, PGNneo may prove to be a useful tool for cancer researchers and clinicians.

## 5. Conclusions

In this study, we developed a proteogenomics-based pipeline to predict neoantigens in noncoding regions, namely, PGNneo. PGNneo is a pipeline that integrates state-of-the-art computational tools. It mainly includes four modules: (1) noncoding somatic variant calling and HLA typing; (2) peptide extraction and customized database construction; (3) variant peptide identification; (4) neoantigen prediction and selection. PGNneo can be easily applied to RNA-seq data and MS data drawn from patients of different cancer types. In summary, PGNneo can specifically detect the neoantigens generated by noncoding regions in tumors, providing guidance for cancer types with a low TMB in coding regions. PGNneo, together with our previous tool, can improve the identification of coding and noncoding region-derived neoantigens and will contribute to a more complete understanding of the tumor immune landscape. This capability holds promise for broadening the repertoire of candidates for therapeutic cancer vaccination and T cell-based therapy and may ultimately extend the neoantigen clinical benefits of immunotherapy.

## Figures and Tables

**Table 1:** Summary of the tools available in PGNneo.

Module	Software	Function
(1) Noncoding somatic variant calling and HLA typing	Trimmomatic-0.39 [30]	Trims adapters and filters low-quality reads
	BWA-0.7.17 [31]	Sequence alignment
	SAMtools(V1.7) [32]	Converts .sam files to .bam, sort, and index files
	GATK4.2.0.0 [34]	Call somatic mutation
	Picard-2.23.9 [33]	Modifies the headers of .bam files
	OptiType-1.3.5 [35]	Predicts HLA typing
(2) Peptide extraction and customized database construction	Annovar [37]	Mutation annotation
	Bedtools(v2.29.2) [38]	Sources the nucleotide sequence
(3) Variant peptide identification	MaxQuant [47]	Peptide identification
(4) Neoantigen prediction and selection	NetMHCpan-4.1 [42]	Calculates the binding affinity of peptides to patient-specific HLA alleles
	Blast-2.11.0+ [45]	Sequence similarity analysis

**Table 2:** Strongly bound neoantigens that are generated by frequently mutated genes in HCC.

Gene	Neoantigen	HLA	%Rank	Bind Level
WWP1	VSHDGATAL	HLA-C*03:04	0.009	SB
NFE2L2	KTDAQAISL	HLA-C*04:03	0.025	SB
TP53	TMAGQLLHV	HLA-A*02:06	0.052	SB
NFE2L2	SSRPAWPTR	HLA-A*33:03	0.08	SB
KMT2D	QQKNPSLFL	HLA-B*13:02	0.126	SB
NFE2L2	GQHSETPSL	HLA-B*15:01	0.154	SB
NFE2L2	WPGHQFFKY	HLA-B*35:01	0.155	SB
KMT2C	IVSSRFCTR	HLA-A*31:01	0.167	SB
KMT2D	QQKNPSLFLI	HLA-B*13:02	0.185	SB
NFE2L2	GIWPGHQFF	HLA-B*15:25	0.206	SB
NFE2L2	LFFETRSRF	HLA-A*24:02	0.226	SB
ATM	AEAGEPLEP	HLA-B*40:06	0.232	SB
WWP1	YRCVPPHPANF	HLA-C*06:02	0.258	SB
KMT2C	KLGDNHFFM	HLA-A*02:01	0.293	SB
NFE2L2	ATRTGRLWWR	HLA-A*31:01	0.323	SB
NFE2L2	HPKSKQISCTW	HLA-B*58:01	0.362	SB
NFE2L2	IWPGHQFF	HLA-A*24:02	0.376	SB
KMT2D	QKNPSLFLI	HLA-B*13:02	0.379	SB
NFE2L2	RMPVIQAAW	HLA-A*24:20	0.385	SB
NFE2L2	RMPVIQAAW	HLA-B*58:01	0.396	SB
WWP1	FSCLSLSGGW	HLA-B*58:01	0.432	SB
ATM	RACQRQAVGIK	HLA-A*30:01	0.433	SB
NFE2L2	KTDAQAISL	HLA-C*03:04	0.442	SB
NFE2L2	GQHSETPSLLK	HLA-A*11:01	0.46	SB
TP53	ATMAGQLLHV	HLA-A*02:06	0.474	SB
WWP1	VSHDGATAL	HLA-C*04:03	0.48	SB

## Data Availability

The noncoding mutation dataset for the lncRNA-seq of HCC samples is available in the Supplementary Materials. The proteomics datasets of the HCC cell line were obtained from the ProteomeXchange Consortium (http://proteomecentral.proteomexchange.org) with the identifier, PXD000529. Another HCC cohort associated with Jiang et al. [28], including RNA-seq files and MS raw data from 10 patients, was downloaded from the Gene Expression Omnibus (GEO) (accession number GSE124535) and iProX database (http://www.iprox.org, accession number IPX0000937000), respectively. The CRC cohort [29], including RNA-seq data and MS raw data from three CRC cell lines and RNA-seq data from one normal fetal small intestine cell line, were downloaded from the GEO (accession number GSE195985) and ProteomeXchange Consortium (identifier PXD028309), respectively.

## Supplementary Materials

The following supporting information can be downloaded at: https://www.mdpi.com/article/10.3390/cells12050782/s1, Figure S1: Results of key steps in neoantigen prediction based on PGNneo for each sample; Table S1: Detailed sample information of the HCC_HF cohort and HCC_HT cohort; Table S2: Noncoding mutations in the HCC_HF cohort; Table S3: Noncoding mutations in the HCC_HT cohort; Table S4: Candidate neoantigens in the HCC_HF cohort; Table S5: Candidate neoantigens in the HCC_HT cohort; Table S6: High-confidence neoantigens in the HCC_HF cohort; Table S7: High-confidence neoantigens in the HCC_HT cohort; Table S8: HLA alleles of each sample in the HCC_HF cohort and HCC_HT cohort; Table S9: The frequency of HLA alleles and the number of corresponding neoantigens in the HCC_HF cohort and HCC_HT cohort; Table S10: Overlap of noncoding neoantigens and noncoding genes between samples in the HCC_HF cohort; Table S11: Frequently mutated genes associated with HCC and the corresponding neoantigens; Table S12: Detailed results on sample information, noncoding region mutations, HLA typing, candidate neoantigens, and high-confidence neoantigens in the CRC cohort; Table S13: Candidate neoantigens, as predicted by five tools; Table S14: Overlap of candidate neoantigens, as predicted by five tools; User’s Manual: Detailed tutorials for using the PGNneo tool.
