# Supplementary material for: PGNneo: A Proteogenomics-Based Neoantigen Prediction Pipeline in Noncoding Regions
Source: Cells. 2023 Mar 1;12(5):782. doi: 10.3390/cells12050782 (PMC10000440; doi:10.3390/cells12050782)
Supplement: Supplementary file 1 [file cells-12-00782-s001.zip › User's Manual.pdf]

User’s Manual

1. Running environment

PGNneo requires a Linux operation system (centos7) with Python (V3.8), R (V4.0), Perl (V5.16) and Java (V1.7) installed.

2. External reference datasets

In order to run normally, several third-party software such as BWA, GATK, and ANNOVAR need extra databases. Here, we provided these files in the “reference” directory, such as “hg38.fa”. In addition, during annotating genetic variants, ANNOVAR software needs lots of databases including: refGene etc. of hg38, putting them into “humandb”folder for the sake of convenience.

3. Usage of PGNneo

Run the following codes before getting started.

```
cd PGNneo
bash start.sh
pip install numpy==1.18.5
pip install pandas==1.0.5
pip install future==0.18.2
pip install pyomo==5.7.3
pip install pysam==0.16.0.1
pip install matplotlib==3.2.2
```

3.1. Somatic variant calling and HLA typing

3.1.1. Software installation and configuration

(1) BWA

Download and install BWA via anaconda or miniconda:

conda config –add channels bioconda

conda install -c bioconda bwa

(2) SAMtools

Download and install SAMtools via anaconda or miniconda:

conda config –add channels bioconda

conda install -c bioconda samtools

(3) GATK

Download and install gatk via anaconda or miniconda:

conda config –add channels bioconda

conda install -c bioconda gatk4

(4) Picard

Download and install picard via anaconda or miniconda:

conda config –add channels bioconda

conda install -c bioconda picard

(5) Bedtools

Download and install bedtools via anaconda or miniconda:

conda config –add channels bioconda

conda install -c bioconda bedtools

(6) ANNOVAR

Download and install Annovar to PGNneo/biosoft/annovar, then execute the following command (Users with root privileges can ignore the following):

chmod 755 biosoft/annovar/convert2annovar.pl

chmod 755 biosoft /annovar/table_annovar.pl

chmod 755 biosoft /annovar/annotate_variation.pl

chmod 755 biosoft /annovar/coding_change.pl

(7) OptiType

tar -zxvf OptiType.tar.gz

In the 'OptiType' directory edit the script config.ini'.

```
[mapping]

# Absolute path to RazerS3 binary, and number of threads to use for mapping

razers3=/path/to/razers3
threads=16

[ilp]

# A Pyomo-supported ILP solver. The solver must be globally accessible in the
# environment OptiType is run, so make sure to include it in PATH.
# Note: this is NOT a path to the solver binary, but a keyword argument for
# Pyomo. Examples: glpk, cplex, cbc.

solver=glpk
threads=1
```

3.1.2. Samples processing

(1) Sample files placement

Create “rnaseq” folder and store the RNA-seq files of tumor and normal paired samples in fastq format in this folder. In addition, create three result folders named “rna_result”, “mut_result” and “HLAtype”, respectively. Test files can be downloaded via start.sh.

(2) Run scripts

python model1_rnaseq_mutation_hla.py control_name_R1.fastq.gz control_name_R2.fastq.gz case_name_R1.fastq.gz case_name_R2.fastq.gz

eg:

python model1_rnaseq_mutation_hla.py con_R1.fastq.gz con_R2.fastq.gz case_R1.fastq.gz case_R2.fastq.gz

(3) Get result files

The results of RNAseq data preprocessing, call mutation and HLA typing are in the “rna_result”, “mut_result” and “HLAtype” folders, respectively.

3.2 Generation of tumor-specific variant peptides

(1) Run scripts

python model2_mutated_peptides.py control_name_R1.fastq.gz case_name_R1.fastq.gz

eg:

python model2_mutated_peptides.py con_R1.fastq.gz case_R1.fastq.gz

(2) Get result files

The results of the generated mutant peptides are under the “mut_result” folder.

3.3 Database construction and variant peptide identification

3.3.1. Software installation and configuration

(1) Edit the script biosoft/gen_mqpar.py.

Edit lines 25:

```
24 # replace fasta path
25 fasta_path = '/path/to/PGNneo/mut_result/mut_ref_db.fasta'
26 fasta_path = ('<fastaFilePath>' + fasta_path + '</fastaFilePath>')
```

Edit lines 78:

```
71 # ok, instead, name the output folder after the named xml output
72 output_folder = os.path.basename(args.outfile)
73 # remove the .xml, if it exists
74 output_folder = re.sub(r'\.xml', '', output_folder)
75 # remove the beginning "mqpar_", if it exists
76 output_folder = re.sub(r'mqpar_', '', output_folder)
77 # append the scratch folder
78 output_folder = ('/path/to/PGNneo/ms_result' + output_folder)
```

(2) Edit the script model3_MS_filtration.py.

Edit lines 7:

```
6 def handle02_xml():
7     cmd1='python ./biosoft/gen_mqpar.py ./biosoft/labelfree.xml /path/to/PGNneo/ms -o ./biosoft/mqpar.xml -t 6'
8     os.system(cmd1)
```

(3) mono

cd biosoft/mono-6.12.0.90

./configure --prxfix=path/to/biosoft

make && make install

(3) MaxQuant

Download MaxQuant to PGNneo/biosoft/MaxQuant.

notes:

To search the proteomics data, we constructed a customized database for each individual tumor sample, including human reference protein, common contaminant protein sequences in the laboratory (cRAP), and cancer-specific proteomes. The human reference protein and cRAP are under the “reference” file named “ref_uniprot_crap.fasta”.

3.3.2. Data processing

(1) Sample files placement

Create a directory “ms” and input the mass spectrometry files into it. Test files can be

downloaded by start.sh.

(2) Run scripts

python model3_MS_filtration.py

(3) Get result files

The result files will be stored under the “ms_resultmqpar/combined/txt” folder.

3.4 Neoantigen prediction and selection

3.4.1 Software installation and configuration

(1) Download NetMHCpan and Blast and include them in your PATH environment variable.

3.4.1 Data processing

(1) Create the result folder named “preneo”.

(2) Run scripts

python model4_neoantigen_prediction_filtration.py

notes:

Input the HLA types predicted in **3.1** or other types that the user interested in when the system prompts:

"please input an HLA class I allele like 'HLA-A02:01' or multiple alleles like 'HLA-A02:01,HLA-B15:01,HLA-C01:02':".

(3) Get result files

Neoantigen predictions and filtering results will be stored in the “preneo” directory.

4. Required Software Downloads

Some of the third-party software needed in PGNneo has been downloaded and placed in the “biosoft” directory, while others need to be downloaded and installed by the user. You need to make sure that each software is available and installed in the correct path. The software download path is shown in Table 1.

Table 1. Summarizes the needed software and download links

Software	Download address
Trimmomatic-0.39[1]	http://www.usadellab.org/cms/index.php?page=trimmomatic
BWA-0.7.17[2]	http://bio-bwa.sourceforge.net/
SAMtools(V1.7)[3]	https://github.com/SAMtools
GATK4.2.0.0[4]	https://software.broadinstitute.org/gatk/download/
Picard-2.23.9[5]	https://broadinstitute.github.io/picard/
Annovar[6]	http://annovar.openbioinformatics.org/en/latest/user-guide/download/
Bedtools(v2.29.2)[7]	https://github.com/arq5x/bedtools2
OptiType-1.3.5[8]	https://github.com/FRED-2/OptiType
NetMHCpan-4.1[9]	http://www.cbs.dtu.dk/services/NetMHCpan-4.1/
MaxQuant[10]	http://www.coxdocs.org/doku.php?id=MaxQuant:start
Blast-2.11.0+[11]	https://ftp.ncbi.nlm.nih.gov/blast/executables/blast+/2.11.0/

Reference:

[1] BOLGER A M, LOHSE M, USADEL B. Trimmomatic: a flexible trimmer for Illumina sequence data [J]. Bioinformatics (Oxford, England), 2014, 30(15): 2114-20.

[2] LI H, DURBIN R. Fast and accurate short read alignment with Burrows-Wheeler transform [J]. Bioinformatics (Oxford, England), 2009, 25(14): 1754-60.

[3] LI H, HANDSAKER B, WYSOKER A, et al. The Sequence Alignment/Map format and SAMtools [J]. Bioinformatics (Oxford, England), 2009, 25(16): 2078-9.

[4] MCKENNA A, HANNA M, BANKS E, et al. The Genome Analysis Toolkit: a MapReduce framework for analyzing next-generation DNA sequencing data [J]. Genome research, 2010, 20(9): 1297-303.

[5] “Picard Toolkit.” Broad Institute, GitHub Repository. https://broadinstitute.github.io/picard/; Broad Institute [J]. 2019,

[6] WANG K, LI M, HAKONARSON H. ANNOVAR: functional annotation of genetic variants from high-throughput sequencing data [J]. Nucleic acids research, 2010, 38(16): e164-e.

[7] QUINLAN A R, HALL I M. BEDTools: a flexible suite of utilities for comparing genomic features [J]. Bioinformatics (Oxford, England), 2010, 26(6): 841-2.

[8] SZOLEK A, SCHUBERT B, MOHR C, et al. OptiType: precision HLA typing from next-generation sequencing data [J]. Bioinformatics (Oxford, England), 2014, 30(23): 3310-6.

[9] REYNISSON B, ALVAREZ B, PAUL S, et al. NetMHCpan-4.1 and NetMHCIIpan-4.0: improved predictions of MHC antigen presentation by concurrent motif deconvolution and integration of MS MHC eluted ligand data [J]. Nucleic acids research, 2020, 48(W1): W449-W54.

[10] TYANOVA S, TEMU T, COX J. The MaxQuant computational platform for mass spectrometry-based shotgun proteomics [J]. Nat Protoc, 2016, 11(12): 2301-19.
[11] MCGINNIS S, MADDEN T L. BLAST: at the core of a powerful and diverse set of sequence analysis tools [J]. Nucleic acids research, 2004, 32(Web Server issue): W20-W5.
