# Supplementary figures and images for: PGNneo: A Proteogenomics-Based Neoantigen Prediction Pipeline in Noncoding Regions
Source: Cells. 2023 Mar 1;12(5):782. doi: 10.3390/cells12050782 (PMC10000440; doi:10.3390/cells12050782)

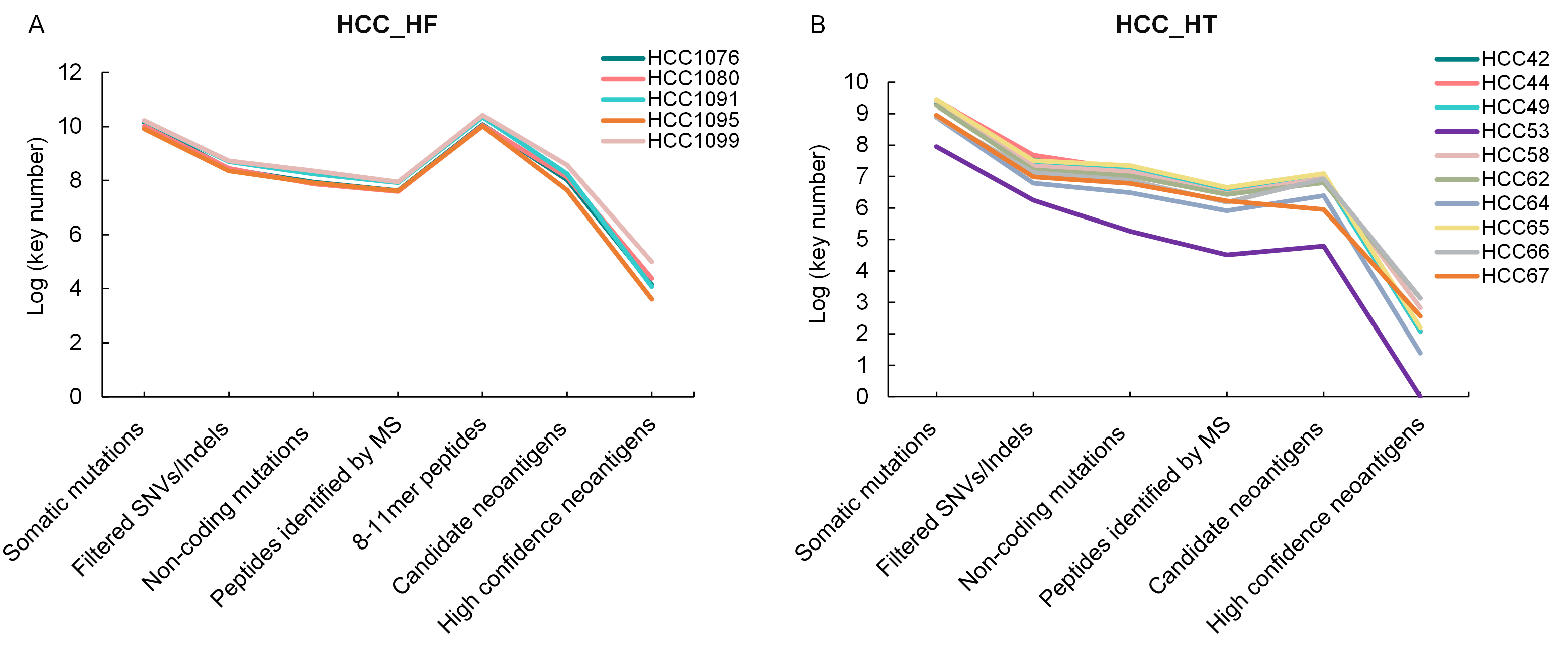

Supplement: Supplementary file 1 [file cells-12-00782-s001.zip › FigS1.tif]
